# New Technique for Impact Calibration of Wide-Range Triaxial Force Transducer Using Hopkinson Bar

**DOI:** 10.3390/s22134885

**Published:** 2022-06-28

**Authors:** Qinghua Wang, Feng Xu, Weiguo Guo, Meng Gao

**Affiliations:** 1 School of Aeronautics, Northwestern Polytechnical University, Xi’an 710072, China; qinghuawang@mail.nwpu.edu.cn (Q.W.); weiguo@nwpu.edu.cn (W.G.); gaomeng891023@163.com (M.G.); 2 School of Astronautics, Northwestern Polytechnical University, Xi’an 710072, China

**Keywords:** triaxial force transducer, wide-range, dynamic calibration, Hopkinson bar, sensitivity matrix

## Abstract

At the current stage, there is an urgent need for new techniques to dynamically calibrate advanced wide-range (up to 10^4^ N~10^5^ N) triaxial force transducers. Based on this background, this paper proposes a novel impact calibration method, specifically for the triaxial force transducer, with a wide range and high-frequency response. In this method, the Hopkinson bar, which is typically used to test the dynamic mechanical properties of materials, was used as a generator to generate reference input force for the transducer. In addition, unlike conventional methods, the transverse sensitivities of the transducer were given necessary importance in the proposed method. The calibration result of the triaxial force transducer was expressed in a sensitivity matrix, containing three main sensitivity coefficients and six transverse sensitivity coefficients. The least squares method (LSM) was used to fit the sensitivity matrix linearly. Calibration experiments were performed on a typical triaxial force transducer. Several key issues, involving the validity and the test range, of the method were further investigated numerically. The feasibility and validity of the method were eventually confirmed. The test range of the method can be up to 10^6^ N.

## 1. Introduction

Triaxial force transducer is a kind of sensing system which can detect and measure force quantity in three-dimensional space [1]. There are significant demands for a triaxial force transducer, having wide range, in many important applications, such as load testing for aircraft landing, crash testing of automobiles, etc. The development of the advanced wide-range triaxial force transducer is receiving increasing attention from researchers in sensors and other related fields [2]. In engineering, the triaxial force transducer needs to be calibrated before it can be put into service. Moreover, the force transducer used for dynamic force measuring ought to be calibrated using a dynamic calibration method, because a transducer calibrated by the static method will have larger measurement error in measuring dynamic forces [3]. However, the dynamic calibration of the wide-range force transducer is currently facing a major challenge in how to excite reference input forces with high amplitudes (10^5^ N level and above) and narrow pulse widths (10^2^ μs level and below).

Currently, the dynamic calibration methods for force transducers can be divided into three types: vibration calibration, impact calibration and step calibration. In the general vibration calibration method [4,5,6,7], the mounting end of the force transducer being calibrated is rigidly connected to a shaker, while the sensitive end of the force transducer is rigidly connected to an external mass. The external mass has the role of generating dynamic (sinusoidal) reference input force for the transducer by oscillating vertically with the shaker. Up to now, the upper limit of the calibration range of vibration calibration facilities can reach 10 kN [8]. In the general impact calibration method [3,9,10,11,12,13,14], an object of known mass is usually made to collide with the force transducer being calibrated. An impulse will be generated during the collision, and it will be input to the transducer as a reference force. Currently available impact calibration facilities can test up to 20 kN, but the pulse width of the generated reference force can only reach the millisecond level [10,11]. In the step calibration method [15], an object of known mass is first suspended above the force transducer being calibrated. Then, by releasing the object, the gravitational force of the object will be suddenly applied on the transducer as a step reference force. The test range of the step calibration device developed in literature [15] can only reach 10^2^ N. 

According to the above, it is difficult to generate a reference input force with an amplitude higher than 20 kN using the dynamic calibration methods available at present. Moreover, it is difficult to generate a reference force with pulse width shorter than the millisecond order using the existing methods. Therefore, the currently available methods cannot cope with the need for dynamic calibration of the advanced force transducer with a range up to 10^5^ N and frequency response up to 10 kHz. On the other hand, in the calibration of multi-dimensional force transducers, the previous studies usually focused only on the main axis sensitivities of the transducer [4,5]. The transverse sensitivities, which may have an influence on measurement accuracy, have not received the attention they deserve. 

In this paper, an impact calibration method specially for the triaxial force transducer, with wide range and high-frequency response, was proposed, based on the Hopkinson bar technique. Calibration experiments were then designed and conducted on a typical wide-range, high-frequency response triaxial force using the newly developed method. The sensitivity of the transducer was expressed in a matrix. The transverse sensitivities of the transducer were evaluated by the off-diagonal elements of this matrix. Finally, several key issues, involving the validity and the test range of the method, were investigated and discussed using the finite element method.

## 2. Theory and Method

### 2.1. Least Squares Method

Similar to the physical quantity of acceleration [16], the force in motion space can be considered as a vector quantity with both magnitude and direction. The physical process of detecting a three-dimensional force in motion space with a triaxial force transducer can be considered mathematically as the process of projecting a three-dimensional vector in force space to a three-dimensional vector in signal space. When the nonlinearities between the inputs and outputs of the transducer are not considered, the projection can be described as:

(1) $$\{\begin{array}{l} U_{X}=S_{XX}F_{X}+S_{XY}F_{Y}+S_{XZ}F_{Z}\\ U_{Y}=S_{YX}F_{X}+S_{YY}F_{Y}+S_{YZ}F_{Z}\\ U_{Z}=S_{ZX}F_{X}+S_{ZY}F_{Y}+S_{ZZ}F_{Z} \end{array}$$

or, in vector form:

(2) $$\left[\begin{array}{c} U_{X}\\ U_{Y}\\ U_{Z} \end{array}\right]=\left[\begin{array}{ccc} S_{XX} & S_{XY} & S_{XZ}\\ S_{YX} & S_{YY} & S_{YZ}\\ S_{ZX} & S_{ZY} & S_{ZZ} \end{array}\right]\left[\begin{array}{c} F_{X}\\ F_{Y}\\ F_{Z} \end{array}\right]$$

The subscripts X, Y and X represent the three sensitive axes of the transducer. The value $U_{i}\left(i=X,\;Y,Z\right)$ represents the output voltage of the i-axis, $F_{i}\left(i=X,\;Y,Z\right)$ represents the reference input force of the i-axis and $S_{ij}\left(i,j=X,\;Y,Z\right)$ represents the sensitivity coefficient of the transducer, where the subscript i denotes the output axis, and the subscript j denotes the input axis. In particular, when i=j, $S_{ii}$ is referred to as a main axis sensitivity; besides, when i≠j, $S_{ij}$ is referred to as a transverse sensitivity [16].

Considering *n* sets of linearly independent inputs and outputs of the triaxial force transducer, then Equation (2) becomes:

(3) $$\left[\begin{array}{ccc} U_{X1} & \cdots & U_{Xn}\\ U_{Y1} & \cdots & U_{Yn}\\ U_{Z1} & \cdots & U_{Zn} \end{array}\right]=\left[\begin{array}{ccc} S_{XX} & S_{XY} & S_{XZ}\\ S_{YX} & S_{YY} & S_{YZ}\\ S_{ZX} & S_{ZY} & S_{ZZ} \end{array}\right]\left[\begin{array}{ccc} F_{X1} & \cdots & F_{Xn}\\ F_{Y1} & \cdots & F_{Yn}\\ F_{Z1} & \cdots & F_{Zn} \end{array}\right]$$

or, in general matrix form:

(4) U=SF

where F (3×n) is the matrix for the reference input force of the transducer. U (3×n) is the matrix for the output voltage of the transducer. S (3×3) is the sensitivity matrix of the transducer. The diagonal elements in S are the main sensitivity coefficients of transducer. The off-diagonal elements in S are the transverse sensitivity coefficients of transducer.

In fact, the relationship from the input to the output of a multi-dimensional force transducer is generally not purely linear [17,18], which leads to a certain deviation from the actual output to its linear regression. Let

(5) $$\epsilon =\left[\begin{array}{ccc} \epsilon_{X1} & \cdots & \epsilon_{Xn}\\ \epsilon_{Y1} & \cdots & \epsilon_{Yn}\\ \epsilon_{Z1} & \cdots & \epsilon_{Zn} \end{array}\right]$$

be the deviation matrix of the triaxial force transducer, then Equation (3) will become:

(6) $$\left[\begin{array}{ccc} U_{X1} & \cdots & U_{Xn}\\ U_{Y1} & \cdots & U_{Yn}\\ U_{Z1} & \cdots & U_{Zn} \end{array}\right]=\left[\begin{array}{ccc} S_{XX} & S_{XY} & S_{XZ}\\ S_{YX} & S_{YY} & S_{YZ}\\ S_{ZX} & S_{ZY} & S_{ZZ} \end{array}\right]\left[\begin{array}{ccc} F_{X1} & \cdots & F_{Xn}\\ F_{Y1} & \cdots & F_{Yn}\\ F_{Z1} & \cdots & F_{Zn} \end{array}\right]+\left[\begin{array}{ccc} \epsilon_{X1} & \cdots & \epsilon_{Xn}\\ \epsilon_{Y1} & \cdots & \epsilon_{Yn}\\ \epsilon_{Z1} & \cdots & \epsilon_{Zn} \end{array}\right]$$

Equation (4) will become:

(7) U=SF+ϵ

Based on Equation (7), the sensitivity matrix S satisfying the least square principle is:

(8) $$S^{*}=\underset{S}{\mathrm{argmin}}{||S||}_{2}^{2}=\underset{S}{\mathrm{argmin}}\epsilon \epsilon^{T}=\underset{S}{\mathrm{argmin}}\left(U-SF\right){\left(U-SF\right)}^{T}$$

$S^{*}$ can be solved according to Equation (9).

(9) $$\frac{\partial \left[\left(U-SF\right){\left(U-SF\right)}^{T}\right]}{\partial S}=0$$

The solution of $S^{*}$ is given in Equation (10).

(10) $$S^{*}=UF^{T}{\left(FF^{T}\right)}^{-1}$$

Actually, what is really required in engineering is the inverse of sensitivity matrix $S^{*}$. As described in Equation (11), the force to be measured is obtained by multiplying the output of the transducer by the inverse of the sensitivity matrix $S^{*}$.

(11) $$F=S^{*\;-1}U$$

### 2.2. Principle of the Hopkinson Bar Method

**Figure 1:**
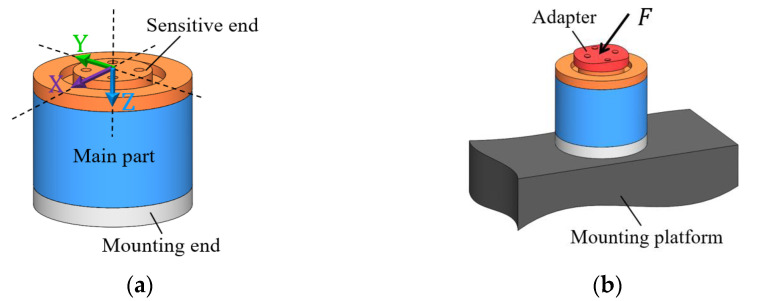
(**a**) Schematic diagram of the typical structure of a triaxial force transducer; (**b**) Schematic diagram of the assembly and the usage of a triaxial force transducer.

Figure 1 depicts the typical structure and the usage of the triaxial force transducers commonly used today. As can be seen, the structure of the transducer can be divided into three parts: the mounting end, the sensitive end and the main part. In practical use, the transducer is held on a platform by its mounting end. A specially designed adapter is usually required to capture and transfer the force to be measured to the sensitive end of the transducer. The sensitive axes of the transducer will then detect the force transferred to the sensitive end and output it as a three-channel voltage signal. The sensing components of transducers are encapsulated in the main part. In this paper, the sensitive axes of the triaxial transducer are represented by X, Y and Z, as shown in Figure 1a.

**Figure 2:**
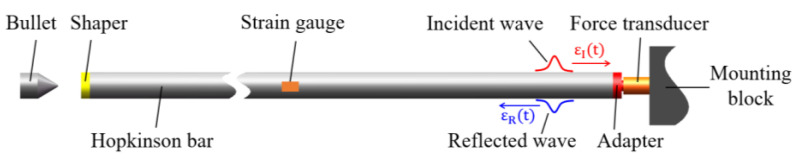
Schematic diagram of the principle of using Hopkinson bar to calibrate force transducer.

The principle of using a Hopkinson bar to calibrate the force transducer is schematically illustrated in Figure 2. Bullets with a taper at its front end are generally adopted to strike the Hopkinson bar. A shaper is attached to the front end of the bar for pulse shaping and to prevent the bar from being damaged by the impact of the bullet. The force transducer being calibrated is mounted on a block. The block constrains the displacement of the transducer in axial direction. The sensitive end (usually requiring an adapter) of the transducer is in good contact with the back end of the bar. The bullet strikes the shaper and excites a compressive wave (the incident wave) in the bar. The wave propagates along the bar to the right and can be detected by the strain gauge glued on the surface of the bar at approximately the middle length. According to the theory of one-dimensional elastic wave propagation, the incident wave will reflect at the interface between the bar and force transducer. The reflected wave can also be detected by the strain gauge. Assuming that the waves propagate along the bar with no dispersion and attenuation, then the strain history on the interface between the Hopkinson bar and transducer can be determined by superimposing the incident and reflected waves as:

(12) $$\varepsilon \left(t\right)=\varepsilon_{I}\left(t\right)+\varepsilon_{R}\left(t\right)$$

where ε(t) is the strain history on the interface, $\varepsilon_{I}\left(t\right)$ and $\varepsilon_{R}\left(t\right)$ are the incident and reflected waves detected by the strain gauge, respectively. Thus, the reference input force of the transducer can be calculated as:

(13) $$F\left(t\right)=EA\left[\varepsilon_{I}\left(t\right)+\varepsilon_{R}\left(t\right)\right]$$

where, E is the elastic modulus of the bar, A is the cross-sectional area of the bar.

**Figure 3:**
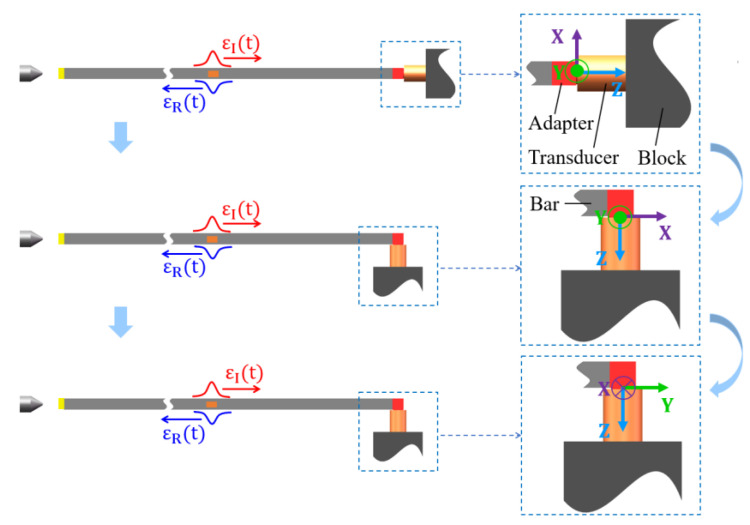
Schematic diagram of the principle of calibrating triaxial force transducer using Hopkinson bar.

For the triaxial force transducer, the principle of its calibration using the Hopkinson bar is shown in Figure 3. The transducer being calibrated was fixed on a mounting block. The block constrained the degrees of freedom of the transducer. A cubic adapter was adopted to capture and transfer the input force for the transducer. The sensitive axes of the triaxial force transducer were tested sequentially by the Hopkinson bar. The attitude of the transducer, as well as the position of the mounting block, had to be changed so that the direction of the force could always be aligned with the direction of the sensitive axis. It is worth noting that in the calibration, shown in Figure 3, only one of the three sensitive axes of the transducer had force input in each test. However, all the three sensitive axes would have voltages output at the same time, due to the coupling between the sensitive axes. Therefore, in this particular case, there would be only one non-zero element in the column vector of the force matrix F in Equation (10), and the other two elements would both be zero. However, the elements in voltage matrix U would all be non-zero elements.

## 3. Experiment

### 3.1. The Triaxial Force Transducer

**Figure 4:**
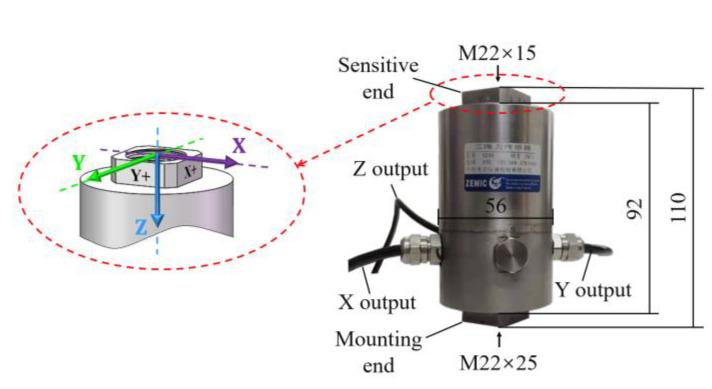
B25B triaxial force transducer from AVIC.

The triaxial force transducer used in this paper was a B25B piezoresistive transducer, as shown in Figure 4, provided by AVIC (Aviation Industry Corporation of China, Ltd., Beijing, China). Table 1 lists the characteristic parameters of the B25B transducer. The main axis sensitivity coefficients of the transducer given in Table 1 were obtained using a static method. The transverse sensitivity coefficients of the transducer were unknown before this paper.

### 3.2. Experiment Set-Ups

**Figure 5:**
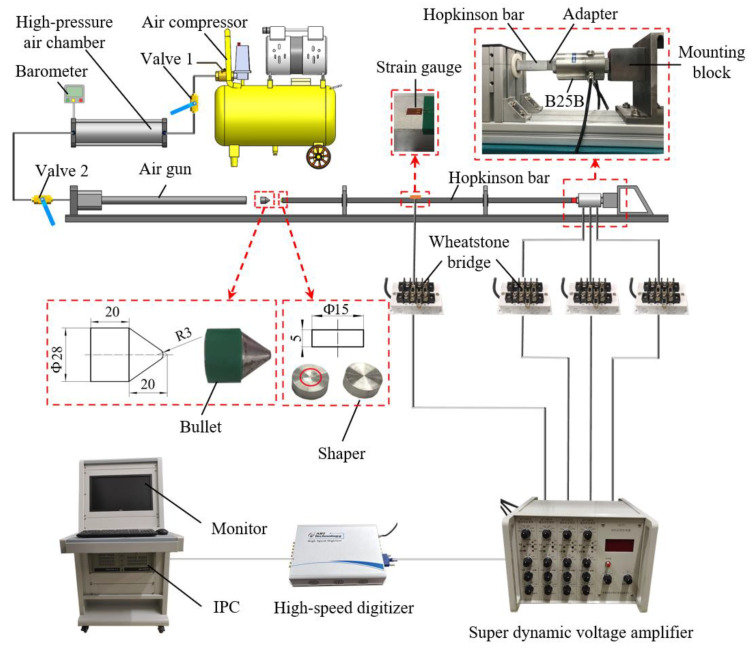
The experiment set-ups based on Hopkinson bar.

The calibration system, established based on the Hopkinson bar, is shown in Figure 5. An air gun was adopted to launch the bullet. When valve 2 was closed and valve 1 was open, the high-pressure air chamber would be inflated by the air compressor. A barometer provided the real-time display of the air pressure in the chamber. When the pressure in the chamber reached an expected value, valve 1 should be closed to stop inflation. At this point, the bullet would be launched by the high-pressure air once valve 2 was opened. The bullet used was made of a 45# high-strength steel. Its geometry and dimensions are given in Figure 5. The velocity of the bullet could be adjusted by changing the pressure in the chamber at the moment of launching. The shapers used were made of a 2024 aluminum alloy. The dimension of the shaper was Φ15×5. The Hopkinson bar used was made of a 7075-aluminum alloy. The bar was a square bar with a section of 20 mm × 20 mm and a length of 1800 mm. The adapter used was a cube with a side length of 20 mm. It was connected rigidly to the transducer with a thread of M22× 15. The adapter, as well as the mounting block and its supporting, were all made of 45# high-strength steel.

The strain gauges used were BE120-3AA strain gauges from AVIC. Two strain gauges were symmetrically glued on the upper and lower surfaces of the bar. These two strain gauges were then connected to a Wheatstone bridge as its two opposing arms. In this way, the stress in the bar would then be converted to a voltage signal by the bridge. The voltage output from the bridge was amplified by a super dynamic voltage amplifier (SDY2107A from BDHSD Co., Ltd., Beidaihe, China; frequency response could be up to 2.5 MHz). The excitation voltages of the transducer were supplied by the power module of the amplifier through the Wheatstone bridge. The voltages output from the transducer were reversely input to the amplifier through the bridge and amplified by the amplifier. Each sensitive axis of the transducer required a signal channel. The voltages output from the amplifier would be captured and recorded by a high-speed digitizer (USB8502, a USB-driven card from ART Technology, Co., Ltd., Beijing, China; sampling frequency up to 40 M/s). The digitizer was driven by an industrial personal computer (IPC). The sampling frequency in experiments was set to 10 M/s. In the experiments, the sensitive axes of the transducer were tested in the order of Z−X−Y.

## 4. Results and Discussion

### 4.1. The Calibration Results

**Figure 6:**
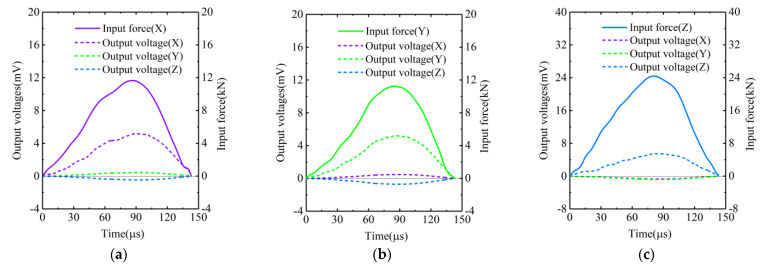
Typical input forces and output voltages of the transducer in the cases where (**a**) the *X*-axis was tested at pressure of 0.15 MPa; (**b**) the *Y*-axis was tested at pressure of 0.15 MPa; (**c**) the *Z*-axis was tested at pressure of 0.3 MPa.

In the experiments, the *Z*-axis of the transducer was tested by the bullet at pressures of 0.05, 0.1, 0.15, 0.2, 0.25, 0.3 and 0.35 MPa. The *X*-axis and *Y*-axis of the transducer were tested at pressures of 0.05, 0.075, 0.1, 0.125, 0.15, 0.175 and 0.2 MPa. The typical input forces and output voltages of the transducer are shown in Figure 6. Specifically, the forces and voltages in Figure 6a were recorded in the case where the *X*-axis was tested at the pressure of 0.15 MPa. The forces and voltages in Figure 6b were recorded in the case where the *Y*-axis was tested at the pressure of 0.15 MPa. The forces and voltages in Figure 6c were recorded in the case where the *Z*-axis was tested at the pressure of 0.3 MPa. It can be seen that the duration of the force pulses excited by the bullet was about 140 μs. In addition, the amplitude of the force pulse increased with the pressure, while the duration of the pulse seemed to be independent of the pressure. On the other hand, it can be seen from Figure 6 that, although only one of the three sensitive axes of transducer had force input, the other two axes would also have voltages output at the same time. This indicated that coupling effects existed between the sensitive axes of the transducer. Moreover, the coupling between *X*-axis and *Y*-axis was positive, and the coupling between *Z*-axis and *X*-axis, as well as the coupling between *Z*-axis and *Y*-axis, were both negative, as illustrated in Figure 6. 

The input force curves and the output voltage curves of the sensitive axes of the transducer had roughly the same trend. This demonstrated that the input forces applied to the transducer could be effectively detected by the sensitive axes. The data used to calculate the sensitivity matrix were the peak values of the force pulses and voltage pulses. The calculation result of the sensitivity matrix of transducer is given in Equation (14). It can be seen that the main axis sensitivity coefficients of the transducer obtained with the dynamic (impact) calibration method were different from those obtained with the static method (Table 1). More specifically, the sensitivity coefficients obtained with the dynamic method were smaller than those obtained with the static method. This coincided with the result of a dynamic evaluation of a single-axis piezoresistive force transducer conducted previously [3].

(14) $$S=\left[\begin{array}{ccc} 0.440 & 0.042 & -0.027\\ 0.037 & 0.460 & -0.032\\ -0.039 & -0.063 & 0.211 \end{array}\right]$$

### 4.2. Discussion

**Figure 7:**
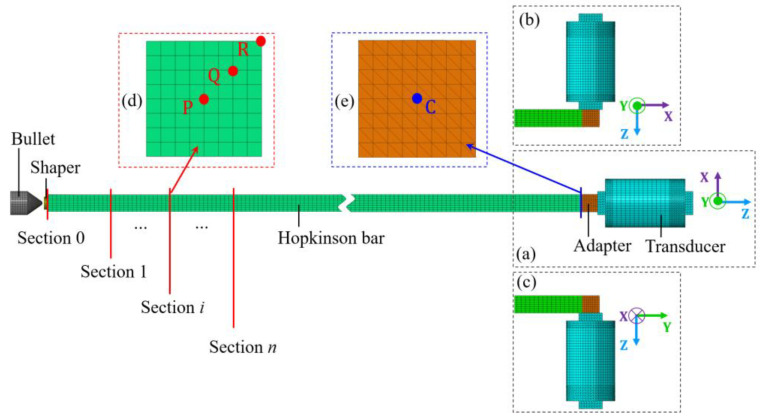
Typical numerical model of the calibration system when (**a**) *Z*-axis; (**b**) *X*-axis; (**c**) *Y*-axis was tested; (**d**) typical cross section of the Hopkinson bar; (**e**) the surface of the adapter which is in contact with the bar.

In this section, the numerical model of the calibration system was built using the commercially available finite element software ADAQUS (Version 2017, Dassault Aviation, Paris, France). A number of simulations were then conducted to investigate the validity and the calibration range of the newly proposed method. The typical model built is shown in Figure 7. Specifically, Figure 7a shows the numerical model, wherein the *Z*-axis was tested. Figure 7b shows the numerical model wherein the *X*-axis was tested. Figure 7c shows the numerical model wherein the *Y*-axis was tested. In addition, a typical cross section of the Hopkinson bar is shown in Figure 7d. The points P, Q, and R were the sampling points on the cross-sections. The surface of the adapter in contact with the bar is shown in Figure 7e. The center point C was the sampling point on this surface.

The materials of the components in numerical simulations were consistent with the settings in experiments. The bullet was assumed to be made of 45# steel. The shaper was assumed to be made of 2024 aluminum alloy. The bar was assumed to be made of 7075-T6 aluminum alloy. The material property constants used in simulations are all listed in Table 2. Since the shaper would deform plastically, due to the impact of bullet, a plastic model should be considered in addition to the elastic model in the material model of the shaper. The plastic model selected for the 2024 aluminum alloy was a Johnson-Cook model developed in literature [19]. The dimensional settings of the components in numerical simulations were also consistent with the settings in experiments. 

The contact properties in the numerical models were all set to frictional contacts with a coefficient of 0.1. The threaded holes at the sensitive end and mounting end of the transducer were simplified into conventional holes with smooth inner surfaces. The main part of the transducer was simplified into a solid elastomer. The threaded bolt of the adapter was simplified into a cylinder with smooth outer surfaces. In the numerical models, the adapter was assembled into the transducer by using a tie constraint. The inner surface of the hole at the mounting end of the transducer was constrained in all the six degrees of freedom to simulate the configuration wherein the transducer was fixed on a mounting block. The type of the tetrahedral elements in the numerical models was set to C3D4 and the type of the hexahedral elements was set to C3D8R. The total number of elements of C3D4 and C3D8R in the numerical model was 127854.

**Figure 8:**
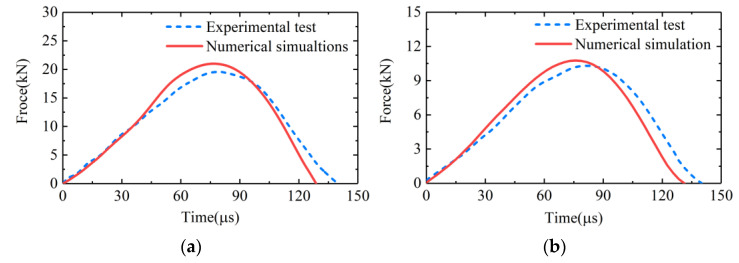
Comparisons of the input forces of the transducer obtained from the experimental tests and the numerical simulations wherein (**a**) the *Z*-axis of the transducer was tested by the bullet at velocity of 20 m/s; (**b**) the *X*-axis of the transducer was tested by the bullet at velocity of 12 m/s.

In order to verify the numerical models, we conducted experimental tests and numerical simulations using the same bullet and at the same impact velocities. Figure 8 shows the comparisons of the input forces of the transducer obtained from the experiments and the numerical simulations. Specifically, the force pulses in Figure 8a were obtained from the experimental test and numerical simulation wherein the *Z*-axis of the transducer was tested at the velocity of 20 m/s. The force pulses in Figure 8b were obtained from the experimental test and numerical simulation wherein the *X*-axis of the transducer was tested at the velocity of 12 m/s. It can be seen that the force pulses obtained from experimental tests and numerical simulations were in good agreement. So, the numerical models built could be considered valid and accurate to simulate the experimental tests. The slight differences between the amplitude and the duration of the force pulses obtained from experiments and simulations might be due to damping and defects of material [20], which were not taken into account in the numerical simulations.

#### 4.2.1. Validation of the Method

**Figure 9:**
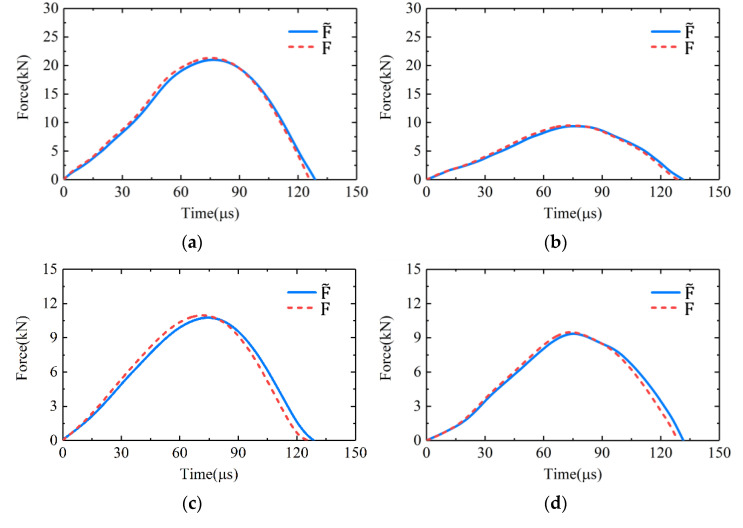
The comparisons of 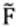 and F in the cases wherein (**a**) the *Z*-axis was tested at the velocity of 20 m/s; (**b**) the *Z*-axis was tested at the velocity of 10 m/s; (**c**) the *X*-axis was tested at the velocity of 12 m/s; (**d**) the *Y*-axis was tested at the velocity of 10 m/s.

According to the principle described in Section 2.2, the input force of the transducer was obtained indirectly from the signal of the strain gauge. Therefore, the validity of the proposed method was predicated on the assumption that the impact force applied to the adapter could be accurately measured by the strain gauge. Let 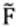 be the input force of the transducer calculated from the strain signals at the midpoint of bar; F be the actual force on the contact surface between bar and adapter, i.e., the actual input force of the transducer. According to the simulations, the stress distribution on the contact surface between bar and adapter could be considered uniform. So, F could be obtained by the product of the stress at point C (see Figure 7) and the cross-sectional area of the bar. The 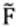 and F were compared in several cases, as shown in Figure 9. It could be seen that the 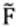 curves were all in very good agreement with the F curves in all the cases. In fact, the relative differences between the durations of the pulses in Figure 9 were less than 2%. The relative differences between the amplitudes of the pulses in Figure 9 were less than 1.5%. It could be considered that the actual input force of the transducer could be accurately measured by the strain signal at the midpoint of the bar. Therefore, the method could achieve the impact calibration of the transducer effectively by generating a measurable reference input force for it. It should be noted that if the structure and usage of the transducer being calibrated are different from Figure 1, the mounting form of the transducer should be changed to ensure the generated reference force can be effectively applied to the sensitive end of the transducer. The method will be valid and applicable, as long as the generated reference load can be effectively applied to the transducer sensitive end and be accurately measured.

#### 4.2.2. Influence of Bullet Geometry 

**Figure 10:**
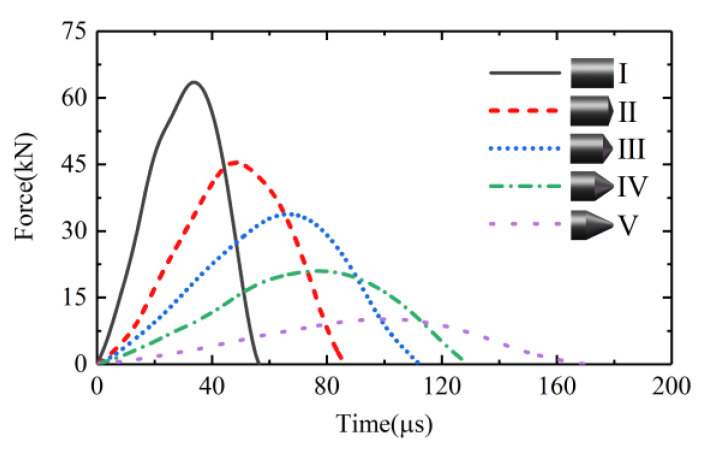
The waveforms of the input forces of *Z*-axis excited by the bullets with various geometries.

Figure 10 shows the waveforms of the input forces of *Z*-axis excited by the bullets with various geometries. The bullets Ι–V in Figure 10 were all 40 mm in length and all had a maximum diameter of 28 mm. The impact velocity of the bullets was 20 m/s. It can be seen that the waveform of the input force was related to the geometry of the bullet. To be more exact, it was related to the taper at the impact end of the bullet. The input force pulse excited by the bullet with a smaller taper at its impact end had a lower amplitude and a wider duration. Yet, the input force pulse excited by the bullet with a larger taper had a higher amplitude and a shorter duration. For an extreme case, the input force pulse excited by bullet Ι, a cylindrical bullet with a maximum taper at its impact end, had the highest amplitude and the shortest duration. 

#### 4.2.3. Influence of Wave Propagation

**Figure 11:**
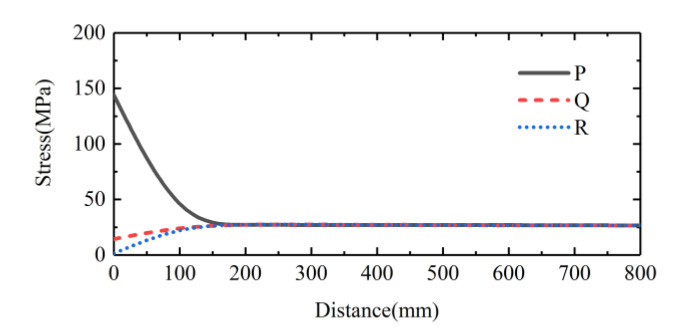
The trends of the $\sigma_{p}$, $\sigma_{q}$ and $\sigma_{r}$ along the bar in the case wherein bullet ΙV (also the bullet in Figure 5) was used.

In order to study the influence of wave propagation, a series of cross sections were selected at 10 cm intervals along the bar, as shown in Figure 7. The P, Q, R points were the sampling points on each section (Figure 7). Let $\sigma_{p}$, $\sigma_{q}$, $\sigma_{r}$ be the stress amplitude at the points P, Q, R, respectively. The values of $\sigma_{p}$, $\sigma_{q}$ and $\sigma_{r}$ on each of the selected sections were recorded. Figure 11 shows the typical trends of the $\sigma_{p}$, $\sigma_{q}$ and $\sigma_{r}$ along the bar. It can be seen the stress distribution on the impact end face of the bar had a significant dispersion. A certain distance was required for the stress to reach a uniform distribution across the section of bar. The distance in Figure 11 was about 160 mm, 8 times the diameter of the bar. Fortunately, the stress wave would propagate with almost a constant amplitude once the stress distribution reached a uniform state. Therefore, the attenuation of the stress amplitude caused by the wave propagation was negligible.

#### 4.2.4. Calibration Range

Building on Equation (13), the reference force input to the transducer could also be described as:

(15) F=Aσ

where A is the sectional area of the bar, σ is the stress on the bar end face in contact with the adapter. According to the principle described in Section 2.2, the deformation of the bar during calibration should be elastic. So, the calibration range of the method was limited by the elastic limit of the bar. Let $\sigma_{e}$ be the elastic limit of the bar, then the input force of the transducer will satisfy the following condition.

(16) $$F<A\sigma_{e}$$

In the present study, the sectional area of the bar was 4×10^−4^ m^2^, the elastic limit of the bar was about 330 MPa. Therefore, the upper limit of the calibration method would be up to 10^6^ N when σ in Equation (15) reached 75.76% of the elastic limit of the bar. By adjusting the velocity and geometry of the bullet, a wide range from 10^4^ N to 10^6^ N could be achieved by the proposed method. 

## 5. Conclusions and Outlook

In this study, a novel impact calibration method applicable for the triaxial force transducer, with wide range and a high-frequency response, was proposed. The Hopkinson bar was used as a force generator in this method. Calibration experiments were conducted on a typical wide-range triaxial force transducer. The transducer sensitivity was expressed in a matrix form to attend simultaneously to main axis sensitivities and transverse sensitivities. The main axis sensitivity coefficients were obtained using the proposed method and were then compared with the coefficients obtained with the static method. Finally, the validity and the test range of the method were investigated and discussed using a numerical method. The conclusions of this study can be summarized as follows:

1. The reference input forces generated by the Hopkinson bar for the sensitive axes of the triaxial force transducer could be accurately measured. The Hopkinson bar is an available and valid force generator for force transducer calibration.
2. The transverse sensitivity coefficients of the triaxial force transducer could also be obtained with the proposed method. The main sensitive coefficients of the transducer obtained using the proposed dynamic method were smaller than those obtained using the static method.
3. The waveform of the reference input force generated by the Hopkinson bar is related to the geometry of the bullet. The bullet with a smaller taper at impact end can generate reference force with a lower amplitude and a wider duration. With a larger taper, the bullet could generate reference force with a higher amplitude and a shorter duration.
4. By adjusting the velocity and geometry of the bullet, the method could achieve a wide calibration range from 10^4^ N to 10^6^ N. The duration of the reference input force generated using the proposed method ranges from 10^1^ μs to 10^2^ μs.

This study provides an effective solution for the calibration of the wide-range triaxial force transducer at this stage. However, the greatest challenge for the calibration of the triaxial force transducer with wide range and high-frequency response is the generating of reference input forces synchronously along the three sensitive axes. This really is a challenge since it is hard to generate measurable high-amplitude force pulses and keep them synchronized within a short duration of tens of or hundreds of microseconds. How to achieve synchronous calibration of the triaxial force transducer deserves great attention from researchers in related fields. This will also be the focus of the authors’ future work. 

## Figures and Tables

**Table 1:** Characteristic parameters of the triaxial force transducer B25B.

Sensitive Axis	Range/kN	Excitation Voltage/V	Natural Frequency/kHz	Sensitivity mV/kN
X	15.0	10.0	12.275	0.480
Y	15.0	10.0	12.275	0.480
Z	30.0	10.0	12.273	0.223

**Table 2:** The material property constants used in the numerical simulations.

Material Parameters	Density ρ$/\mathrm{kg}\times m^{3}$	Elastic Modulus *E*/MPa	Poisson Ratio υ	Johnson-Cook Model			
				A/MPa	B/MPa	*n*	C
45#Steel	7850	210,000	0.30	\	\	\	\
AA7075-T6	2800	71,000	0.33	\	\	\	\
AA2024	2700	70,000	0.33	360	649	0.68	0.0146

## Data Availability

The data that support the findings of this study are available from the corresponding authors upon reasonable request.
